# Loss of Cervical Sympathetic Chain Input to the Superior Cervical Ganglia Affects the Ventilatory Responses to Hypoxic Challenge in Freely-Moving C57BL6 Mice

**DOI:** 10.3389/fphys.2021.619688

**Published:** 2021-04-22

**Authors:** Paulina M. Getsy, Gregory A. Coffee, Yee-Hsee Hsieh, Stephen J. Lewis

**Affiliations:** ^1^Department of Pediatrics, Division of Pulmonology, Allergy and Immunology, Case Western Reserve University, Cleveland, OH, United States; ^2^The Department of Physiology and Biophysics, Case Western Reserve University, Cleveland, OH, United States; ^3^Division of Pulmonary, Critical Care and Sleep Medicine, University Hospitals Case Medical Center, Case Western Reserve University, Cleveland, OH, United States; ^4^Department of Pharmacology, Case Western Reserve University, Cleveland, OH, United States

**Keywords:** cervical sympathetic chain transection, superior cervical ganglion, hypoxic gas challenge, ventilatory parameters, C57BL6 mice

## Abstract

The cervical sympathetic chain (CSC) innervates post-ganglionic sympathetic neurons within the ipsilateral superior cervical ganglion (SCG) of all mammalian species studied to date. The post-ganglionic neurons within the SCG project to a wide variety of structures, including the brain (parenchyma and cerebral arteries), upper airway (e.g., nasopharynx and tongue) and submandibular glands. The SCG also sends post-ganglionic fibers to the carotid body (e.g., chemosensitive glomus cells and microcirculation), however, the function of these connections are not established in the mouse. In addition, nothing is known about the functional importance of the CSC-SCG complex (including input to the carotid body) in the mouse. The objective of this study was to determine the effects of bilateral transection of the CSC on the ventilatory responses [e.g., increases in frequency of breathing (Freq), tidal volume (TV) and minute ventilation (MV)] that occur during and following exposure to a hypoxic gas challenge (10% O_2_ and 90% N_2_) in freely-moving sham-operated (SHAM) adult male C57BL6 mice, and in mice in which both CSC were transected (CSCX). Resting ventilatory parameters (19 directly recorded or calculated parameters) were similar in the SHAM and CSCX mice. There were numerous important differences in the responses of CSCX and SHAM mice to the hypoxic challenge. For example, the increases in Freq (and associated decreases in inspiratory and expiratory times, end expiratory pause, and relaxation time), and the increases in MV, expiratory drive, and expiratory flow at 50% exhaled TV (EF_50_) occurred more quickly in the CSCX mice than in the SHAM mice, although the overall responses were similar in both groups. Moreover, the initial and total increases in peak inspiratory flow were higher in the CSCX mice. Additionally, the overall increases in TV during the latter half of the hypoxic challenge were greater in the CSCX mice. The ventilatory responses that occurred upon return to room-air were essentially similar in the SHAM and CSCX mice. Overall, this novel data suggest that the CSC may normally provide inhibitory input to peripheral (e.g., carotid bodies) and central (e.g., brainstem) structures that are involved in the ventilatory responses to hypoxic gas challenge in C57BL6 mice.

## Introduction

Pre-ganglionic sympathetic neurons emanating from the thoracic spinal cord (T1–T4) course in the left and right cervical sympathetic chains (CSC) and terminate on the cell bodies of post-ganglionic sympathetic neurons in the ipsilateral superior cervical ganglia (SCG) (Rando et al., 1981; Tang et al., 1995a,b,c; Llewellyn-Smith et al., 1998). The majority of post-ganglionic cells leave the SCG via the internal and external carotid nerves (Bowers and Zigmond, 1979; Buller and Bolter, 1997; Asamoto, 2004; Savastano et al., 2010), with the ganglioglomerular nerve (GGN) branching from the external carotid nerve to innervate glomus cells, chemoafferent nerve terminals, and vasculature within the carotid body (Biscoe and Purves, 1967; Zapata et al., 1969; Bowers and Zigmond, 1979; McDonald and Mitchell, 1981; McDonald, 1983; Verna et al., 1984; Torrealba and Claps, 1988; Ichikawa, 2002; Asamoto, 2004; Savastano et al., 2010), and baroafferent nerve terminals within the carotid sinus (Floyd and Neil, 1952; Rees, 1967; Bolter and Ledsome, 1976; Felder et al., 1983; Buller and Bolter, 1993).

The projections of the SCG to a series of inter-related structures, such as the carotid body and carotid sinus, upper airway and tongue (Flett and Bell, 1991; Kummer et al., 1992; O’Halloran et al., 1996, 1998; Hisa et al., 1999; Wang and Chiou, 2004; Oh et al., 2006), and nuclei within the hypothalamus and brainstem, including the nucleus tractus solitarius (nTS) (Cardinali et al., 1981a,b, 1982; Gallardo et al., 1984; Saavedra, 1985; Wiberg and Widenfalk, 1993; Westerhaus and Loewy, 1999; Esquifino et al., 2004; Hughes-Davis et al., 2005; Mathew, 2007), provide evidence that the SCG is a vital integrative structure regulating cardiorespiratory function. Previous studies have shown that electrical stimulation of the CSC decreases arterial blood pressure and upper airway resistance in rats (O’Halloran et al., 1996, 1998). Moreover, there is substantial (and conflicting) evidence as to the ability of sympathetic innervation to the carotid body to influence resting activity of glomus cells and chemoafferents within the carotid sinus nerve (CSN), and modulate changes in activity during hypoxic gas challenge (HXC) (Prabhakar, 1994). For instance, CSC and GGN activity increases during hypoxic challenge, suggesting the release of neurotransmitters, such as norepinephrine, dopamine and neuropeptide Y (Lahiri et al., 1986; Matsumoto et al., 1986, 1987, Yokoyama et al., 2015), and conversely activation of the GGN decreases the hypoxic response of chemosensors within the cat carotid body (McQueen et al., 1989). A variety of responses have also been reported upon application of neurotransmitters released by CSC and GGN nerve terminals (e.g., norepinephrine, dopamine and neuropeptide Y) to *in vivo* and *in vitro* carotid body preparations, including (1) a biphasic pattern consisting of initial brief bursts in CSN activity and then long-lasting inhibition (Bisgard et al., 1979), (2) a biphasic pattern consisting of initial brief decreases in CSN activity and then long-lasting excitation (Matsumoto et al., 1981), (3) indirect excitation of carotid body glomus cells via constriction of arteriolar blood flow in the carotid body (Potter and McCloskey, 1987; Yokoyama et al., 2015), (4) direct activation of glomus cells and/or chemosensory afferents (Matsumoto et al., 1980; Lahiri et al., 1981; Milsom and Sadig, 1983; Heinert et al., 1995; Pang et al., 1999), and (5) direct inhibitory action of glomus cells and/or chemosensory afferents (Zapata et al., 1969; Zapata, 1975; Llados and Zapata, 1978; Mills et al., 1978; Folgering et al., 1982; Kou et al., 1991; Pizarro et al., 1992; Bisgard et al., 1993; Prabhakar et al., 1993; Ryan et al., 1995; Almaraz et al., 1997; Overholt and Prabhakar, 1999).

Wild-type and genetically-engineered mice are widely used to understand the mechanisms by which hypoxic challenges elicit carotid body-*dependent* and -*independent* ventilatory responses (He et al., 2000, 2002, 2003; Kline and Prabhakar, 2000; Pérez-García et al., 2004; Kline et al., 2005; Pichard et al., 2015; Gao et al., 2017; Wang et al., 2017; Ortega-Sáenz et al., 2018; Peng et al., 2018; Prabhakar et al., 2018). The morphology, neurophysiology and neuropharmacology of the mouse CSC-SCG has received considerable investigation over the years (Black et al., 1972; Yokota and Yamauchi, 1974; Banks and Walter, 1975; Inoue, 1975; Lewis and Burton, 1977; Forehand, 1985; Kidd and Heath, 1988; Gibbins, 1991; Kasa et al., 1991; Little and Heath, 1994; Jobling and Gibbins, 1999; El-Fadaly and Kummer, 2003; David et al., 2010; Cadaveira-Mosquera et al., 2012; Pashai et al., 2012; Alberola-Die et al., 2013; Liu and Bean, 2014; Martinez-Pinna et al., 2018; Mitsuoka et al., 2018; Feldman-Goriachnik and Hanani, 2019; Simeone et al., 2019; Rivas-Ramírez et al., 2020). Mouse tissues that receive post-ganglionic projections from the SCG have also been heavily investigated (Krieger et al., 1976; García et al., 1988; Kawaja and Crutcher, 1997; Maklad et al., 2001; Pankevich et al., 2003a,b; Ivanusic et al., 2013; Karlsen et al., 2013; Lindborg et al., 2018; Ziegler et al., 2018; Teshima et al., 2019). Nonetheless, there is no direct evidence that the CSC-SCG complex innervates the carotid bodies of mice, and this is likely on the basis of the presence of sympathetic (i.e., tyrosine-hydroxylase-positive) nerve terminals and adrenergic receptors in these structures (Prieto-Lloret et al., 2007; Roux et al., 2008; Kåhlin et al., 2010; Chai et al., 2011).

Patients with chronic T1–T4 spinal cord injury present with cardiorespiratory disturbances consistent with diminished activity of the CSC-SCG complex (DiMarco et al., 2009; Heutink et al., 2014; Sankari et al., 2014, 2019; Berlowitz et al., 2016; Hachmann et al., 2017; Shin et al., 2019) including, enhanced peripheral (carotid body) chemoreflex sensitivity, which is a primary cause of sleep-disordered breathing in these subjects (Tester et al., 2014; Bascom et al., 2016). In contrast, studies in rats have found that mid-thoracic spinal cord injury is associated with enhanced cardiac sympathetic activity and cardiac sympathetic hyperinnervation that increases the susceptibility to life-threatening arrhythmias (Rodenbaugh et al., 2003; Collins et al., 2006; Lujan and DiCarlo, 2007; Lujan et al., 2009, 2010, 2012, 2014). Studies in rats have also confirmed that hypoactivity of the CSC greatly enhances the likelihood of stroke in hypertensive models (Sadoshima et al., 1981; Sadoshima and Heistad, 1983; Werber and Heistad, 1984).

The effects of bilateral transection of the CSC (CSCX) or bilateral removal of the SCG (SCGX) have been investigated on a variety of physiological functions/variables in the mouse (Krieger et al., 1976; García et al., 1988; Kawaja and Crutcher, 1997; Pankevich et al., 2003a,b; Karlsen et al., 2013; Lindborg et al., 2018; Ziegler et al., 2018). However, to our knowledge, no studies, in any species, have determined the roles of the CSC-SCG complex on ventilatory functions and responses to hypoxic challenges *in vivo*. The aim of this study was to determine the effects of sham-operated (SHAM) and bilateral CSCX (performed 4 days before testing) on resting ventilatory parameters in freely-moving adult male C57BL6 mice, and on their ventilatory responses to HXC using whole-body plethysmography as described previously (Palmer et al., 2013a,b; Gaston et al., 2014; Getsy et al., 2014; Palmer et al., 2015).

The C57BL6 mouse has proven to be an invaluable murine model in which to investigate the physiological systems involved in ventilatory control processes (Tankersley et al., 1994, 2000), and this strain is used widely to generate genetic knock-out mutants to investigate the molecular mechanisms underlying the responses of mice to hypoxic and/or hypercapnic gas challenges (Kline and Prabhakar, 2000, Kline et al., 2005; Palmer et al., 2013a). The ventilatory responses that will be described in the SHAM mice (during and following the HXC) were consistent with previous studies from our laboratory (Palmer et al., 2013a,b, 2015; Gaston et al., 2014; Getsy et al., 2014). Our Getsy et al. (2014) manuscript provides a detailed set of analyses of the ventilatory responses that occur in C57BL6, Swiss Webster and B6AF1 mice before, during, and after a brief HXC. We found that (1) the HVR in C57BL6 mice consists of an initial increase in frequency of breathing (Freq) followed by substantial decline (roll-off) toward pre-HXC values, whereas the Freq responses in Swiss Webster and B6AF1 mice were robust with minimal roll-off, and (2) the post-HXC (i.e., return to room-air) responses consisted of a rapid and sustained rise in Freq in C57BL6 mice, a sustained rise in the B6AF1 mice, which is known as a form of short-term potentiation (STP) (Powell et al., 1998), and a gradual return to pre-hypoxic challenge levels in the Swiss-Webster mice.

The ways and mechanisms by which activation of sympathetic nerves affects carotid body function under normoxic and hypoxic conditions have received considerable attention (Overholt and Prabhakar, 1999). For instance, compelling evidence shows that sympathetic nerve terminals innervate glomus cells and the microvasculature within the carotid bodies (Vázquez-Nin et al., 1978; McDonald, 1983; Verna et al., 1984), and that norepinephrine is the major neurotransmitter released by these terminals (Almaraz et al., 1997). The roles of norepinephrine and dopamine and α_1_-, α_2_-, and β-adrenoceptors and dopamine receptors have also been extensively studied. It is apparent that activation of sympathetic nerves can induce a multiplicity of effects within the carotid body. First, activation of the sympathetic nerves indirectly activate glomus cells by constricting arterioles (by activation α_1_-adrenoceptors and dopamine receptors) in the carotid body, effectively resulting in a hypoxic environment for glomus cells (Llados and Zapata, 1978; Majcherczyk et al., 1980; Matsumoto et al., 1981; Yokoyama et al., 2015). In addition, co-release of neuropeptide Y from sympathetic nerve terminals also reduces blood flow within the carotid bodies (Potter and McCloskey, 1987). In addition, activation of β-adrenoceptors and dopamine receptors on glomus cells directly activates these cells (Eldridge and Gill-Kumar, 1980; Lahiri et al., 1981; Gonsalves et al., 1984). On the other hand, intra-carotid artery infusions of norepinephrine depress resting chemoreceptor activity and also attenuate hypoxic excitation of the carotid body, effects mediated by α_2_-adrenoceptors (Kou et al., 1991; Pizarro et al., 1992; Prabhakar et al., 1993; Almaraz et al., 1997). Moreover, endogenous norepinephrine indirectly inhibits carotid body chemoafferent activity (Overholt and Prabhakar, 1999) via inhibition of glomus cell activity (presumably the release of excitatory neurotransmitters) by decreasing the magnitude and rate of macroscopic Ca^2+^ current influx (Overholt and Prabhakar, 1999). As such, it is likely to question how the absence of functional sympathetic input to the carotid bodies and other structures controlling ventilatory processes, would affect baseline parameters and the responses to HXC.

As mentioned, it has been established that HXC increases neural activity in the CSC and GGN (Lahiri et al., 1986; Matsumoto et al., 1986, 1987). As such, it is proposed that HXC activates a carotid sinus (chemoafferent) nerve-brainstem-descending spinal cord pathway that increases pre-ganglionic sympathetic nerve activity, which in turn activates post-ganglionic neurons in the SCG, including those projecting to the carotid body via the GGN. It should be noted that there is compelling evidence that SCG cells are not directly responsive to hypoxia (Rigual et al., 1999; Nunes et al., 2012; Buckler and Turner, 2013; Gao et al., 2019; Bernardini et al., 2020), although there is equally compelling evidence that SCG cells [and in particular small intensely fluorescent (SIF) cells] are hypoxia-sensitive (Hanson et al., 1986; Brokaw and Hansen, 1987; Dinger et al., 1993; Strosznajder, 1997; Nunes et al., 2010, 2012).

One principal finding of this study is that transection of the CSC is not equivalent to bilateral removal of the SCG (see Conclusion), suggesting multiple effects of hypoxia on neural signaling within the CSC-SCG pathway.

## Materials and Methods

### Permissions

All studies were carried out in accordance with the National Institutes of Health “Guide for the Care and Use of Laboratory Animals” (NIH Publication No. 80-23) revised in 1996. The protocols were approved by the Animal Care and Use Committee of Case Western Reserve University.

### Animals

C57BL6 male mice were purchased from Jackson Laboratory (Bar Harbor, ME, United States). Mice were delivered pathogen free, and housed under specific-pathogen free conditions with a 12 h light-dark cycle. All procedures were performed in accordance with the National Institute of Health (NIH) guidelines for care and use of laboratory animals^1^ and were approved by the Institutional Animal Care and Use Committee at Case Western Reserve University.

### Cervical Sympathetic Chain Transection (CSCX)

**FIGURE 1:**
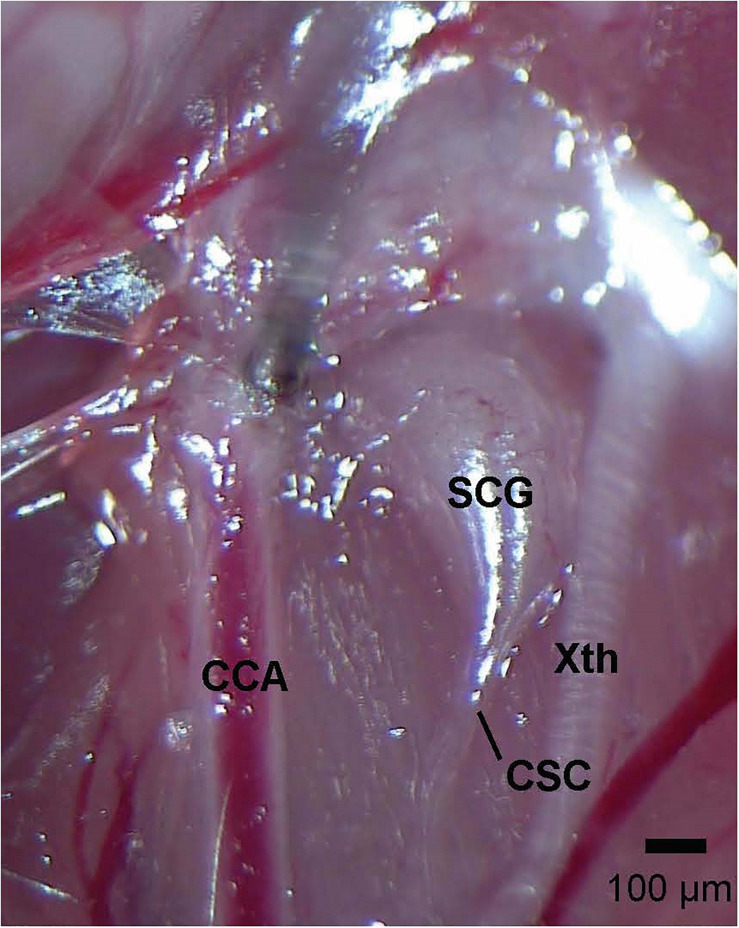
Photograph of the left cervical sympathetic chain (CSC) and left superior cervical ganglion (SCG) of an adult C57BL6 mouse. The vagus nerve (Xth) and common carotid artery (CCA) are also shown. The scale bar is 100 μm.

Adult mice (12 weeks) were anesthetized with an intraperitoneal injection of ketamine (80 mg/kg, Ketaset, Zoetis, Parsippany, NJ, 100 mg/ml solvent) and xylazine (10 mg/kg, Akorn Animal Health, Lake Forest, IL, United States, 20 mg/ml solvent), and placed on a surgical station allowing body temperature to be maintained at 37°C via a heating pad (SurgiSuite, Kent Scientific Corporation, Torrington, CT, United States). The adequacy of anesthesia was regularly checked by nociceptive stimulus (e.g., a toe pinch). The SCG-CSC was identified behind the carotid artery bifurcation (Figure 1), and the CSC was cut using micro-scissors approximately 1 mm from the point where the CSC enters the SCG. In SHAM mice, the SCG-CSC was identified but not cut. The mice were allowed 4 days to recover from surgery. This time-point for recovery was chosen based on evidence that catecholamine levels in the carotid bodies, identified by tyrosine hydroxylase positive nerve terminals, are markedly reduced 3–4 days after removal of the ipsilateral SCG (Mir et al., 1982; González-Guerrero et al., 1993; Ichikawa and Helke, 1993; Ichikawa, 2002). All mice were monitored for pain and distress every day following surgery. Mice were given an injection of the non-steroidal anti-inflammatory drug, carprofen (2 mg/kg, IP), 24 and 48 h post-surgery to reduce any pain or inflammation at the incision site. None of the mice showed any signs of pain or inflammation from the surgeries and began moving about the cages and eating and drinking approximately 1 h after surgery. Mice were weighed daily to ensure proper weight gain. We have determined that these injections of carprofen do not affect resting ventilation or the response to HXC on day 4 post-surgery (data not shown).

### Whole-Body Plethysmography

On the day of study, mice were placed in individual whole-body plethysmographs (Buxco^®^ Small Animal Whole Body Plethysmography, DSI a division of Harvard Biosciences, Inc., St. Paul, MN, United States) to continuously record ventilatory parameters in the freely-moving state, as described in detail previously (Palmer et al., 2013a,b, 2015; Gaston et al., 2014; Getsy et al., 2014). The whole body plethysmography experiments were done by investigators (P.M.G, G.A.C, or Y-H.H) who were blinded to which surgery the mouse had undergone. The protocols were designed to ensure that the studies were done in a controlled fashion. More specifically, sub-groups of SHAM (*n* = 2) and CSCX (*n* = 2) mice were studied on the same day. The studies therefore took a total of 6 days over a 3 weeks period to study 11 SHAM and 12 CSCX mice. Because of the experimental paradigm, the collected data are likely to accurately define the effects of CSCX on resting ventilatory parameters and the response to HXC. The array of ventilatory parameters were chosen to provide an in-depth analysis of the differences in resting breathing patterns and responses to the hypoxic gas challenge in SHAM and CSCX mice (Solberg et al., 2006; Moore et al., 2012; Palmer et al., 2013a,b, 2015; Gaston et al., 2014; Getsy et al., 2014; Villiere et al., 2017). Directly recorded parameters were (a) Freq, (b) tidal volume, (TV), (c) inspiratory time (Ti, duration of inspiration) and expiratory time (Te, duration of expiration), (d) end inspiratory pause (EIP, pause between end of inspiration and start of expiration) and end expiratory pause (EEP, pause between end of expiration and start of inspiration), (e) expiratory flow at 50% expired TV (EF_50_), (f) peak inspiratory flow (PIF) and peak expiratory flow (PEF), (g) relaxation time (decay of respiration to 36% of maximum PIF), (h) relative rate of achieving PEF (Rpef), and (i) rejection index (RI, % of non-eupneic breaths such as apneas per epoch). Calculated parameters were (a) minute ventilation (MV, Freq × TV), (b) Ti/Te and Ti/(Ti + Te), (c) PIF/PEF, (d) inspiratory drive (TV/Ti) and expiratory drive (TV/Te), (e) rejection index corrected for respiratory frequency (RI/Freq), and (f) the number of apneic pauses per epoch (Te/RT)-1. With respect to the rejection index, the plethysmography system included a rejection algorithm that was set to reject breaths that did not reflect normal TV breathing, and as such rejected (a) abnormal breaths (abnormal balance of inspiratory and expiratory volumes), apneas, sighs, post-sighs, sniffs, and waveforms that most likely arose from activities such as grooming the face, hands, and rear (Getsy et al., 2014). Minimum TV was set at 0.05 ml, minimum Ti was set at 0.04 s, maximum Te was set at 0.5 s, and the ratio of inspiratory volume/expiratory volume (i.e., volume balance) was set to a range of 90–110%. Values were rejected when they fell outside the above criteria and when (a) Ti was greater than 2 times Te, (b) PIF could not be distinguished from PEF, and (c) EF_50_, Rpef, or RT could not be computed (Getsy et al., 2014). All directly recorded parameters (i.e., Freq, TV, MV, Ti, Te, EIP, EEP, PIF, PEF, Rpef, relaxation time and rejection index) were extracted from the raw waveforms using proprietary Biosystem XA and FinePointe software (Data Sciences International, St. Paul, MN, United States) as described previously for mice (Palmer et al., 2013a,b; Gaston et al., 2014; Getsy et al., 2014) and as detailed in the Data Sciences International/Buxco website reference to parameters provided by FinePointe Software using whole-body plethysmography^2^. Data was extracted as individual data points (e.g., a Freq value for a particular 15 s epoch) and placed in excel spreadsheets.

### Protocols for Hypoxic Gas Challenge

SHAM and CSCX mice were placed in the plethysmography chambers and allowed approximately 60 min to settle to allow for resting parameters to reach stable levels before the freely-moving mice were exposed to a 5 min HXC (10% O_2_ and 90% N_2_) and then re-exposed to room-air for 15 min.

### Statistics

A data point before (15 min), during (5 min) and after (15 min) HXC was collected every 15 s. To determine the total responses (cumulative % changes from pre-HXC values) during HXC and return to room-air for each mouse, we summed the values recorded before and during the challenge and those upon return to room-air. Regarding the pre-HXC (baseline) phase, the breaths for each 15 s epoch were averaged over the last 5 min of the entire 15 min baseline recording period resulting in 20 values for each mouse that was averaged to give the resting value for each mouse. The mean and SEM for the 11 SHAM and 12 CSCX mice was then derived from the individual values. Similarly, twenty 15 s epoch values were derived during the 5 min hypoxic challenge and sixty 15 s epoch values were derived for the post-hypoxia (room-air) phase. Again, the mean and SEM for the 11 SHAM and 12 CSCX mice was derived from the individual values for the HXC and room-air phases under examination. We then determined the cumulative response for each mouse by the formulas, (a) total HXC response = (sum of the 20 values during HXC) − (mean of the pre-HXC values × 20), and (b) total room-air response = (sum of the 60 values during room-air phase) − (mean of the pre-HXC values × 60). We then determined the mean and SEM of the group data. We also calculated the total responses during the HXC 0–105 sec epoch and 106–300 sec epoch, in addition to the entire HXC 5 min (0–300 sec epoch). All data are presented as mean ± SEM. All data were analyzed by one-way or two-way ANOVA followed by Student’s modified *t*-test with Bonferroni corrections for multiple comparisons between means (Palmer et al., 2013a,b).

## Results

### Resting Parameters

A summary of the mouse descriptors and resting ventilatory parameters is provided in Table 1. There were 11 mice in the SHAM group and 12 mice in the CSCX group. The ages and body weights of the two groups were similar to one another (*P* > 0.05, for both comparisons). Accordingly, no corrections for body weights were applied to the ventilatory data pertaining to volumes (e.g., TV and peak inspiratory and expiratory flows). There were no between-group differences for any recorded or calculated ventilatory parameter (*P* > 0.05, for all comparisons). In the following Figures 3–10, the left panel of each row will show the actual values recorded before, during the 5 min hypoxic (10% O_2_ and 90% N_2_) challenge, and upon to room-air. The middle panels of each row will show the arithmetic change recorded over the first 60 s of hypoxic exposure. The right panels of each row will show the total response (% change from pre) during three epochs of the 5 min (300 s) hypoxic challenge, namely 0–105, 106–300, and 0–300 s. These epochs were those which best represented the dramatic differences between SHAM and SCGX mice (see Conclusion).

**TABLE 1:** Baseline parameters in sham-operated (SHAM) mice and in mice with bilateral transection of the cervical sympathetic chain (CSCX).

**Parameter**	**SHAM**	**CSCX**
Number of mice	11	12
Age, days	101 ± 1	102 ± 1
Body weight, grams	27.2 ± 0.5	27.5 ± 0.8
Frequency (breaths/min)	196 ± 5	188 ± 6
Tidal volume (TV, ml)	0.149 ± 0.007	0.157 ± 0.014
Minute ventilation (ml/min)	28.9 ± 1.1	28.6 ± 1.8
Inspiratory time (Ti, sec)	0.112 ± 0.003	0.114 ± 0.003
Expiratory time (Te, sec)	0.207 ± 0.006	0.220 ± 0.009
End inspiratory pause (EIP, msec)	2.90 ± 0.08	2.85 ± 0.07
End expiratory pause (EEP, msec)	34.5 ± 3.8	32.7 ± 6.1
Ti/Te	0.551 ± 0.016	0.526 ± 0.015
Ti/(Ti + Te)	0.353 ± 0.007	0.343 ± 0.006
Peak inspiratory flow (PIF, ml/sec)	2.38 ± 0.09	2.44 ± 0.16
Peak expiratory flow (PEF, ml/sec)	1.51 ± 0.06	1.42 ± 0.09
PIF/PEF	1.59 ± 0.05	1.74 ± 0.07
Expiratory flow at 50% exhaled TV (EF_50,_ ml/sec)	0.072 ± 0.004	0.071 ± 0.003
Relaxation time (RT, sec)	0.100 ± 0.003	0.103 ± 0.004
Rate of achieving PEF (Rpef)	0.138 ± 0.13	0.166 ± 0.019
Inspiratory drive (TV/Ti, ml/sec)	1.36 ± 0.06	1.38 ± 0.09
Expiratory drive (TV/Te, ml/sec)	0.72 ± 0.03	0.70 ± 0.04
Rejection index (RI, %)	15.4 ± 1.9	15.2 ± 1.9
(Rejection index/frequency) × 100	7.9 ± 0.9	8.3 ± 1.2
Apneic pauses (Te/RT)-1	1.07 ± 0.03	1.15 ± 0.05

*The data are presented as mean ± SEM. There were no between-group differences for any parameter (P > 0.05, for all comparisons).*

### Hypoxic Challenges – Frequency of Breathing, Tidal Volume and Minute Ventilation

**FIGURE 2:**
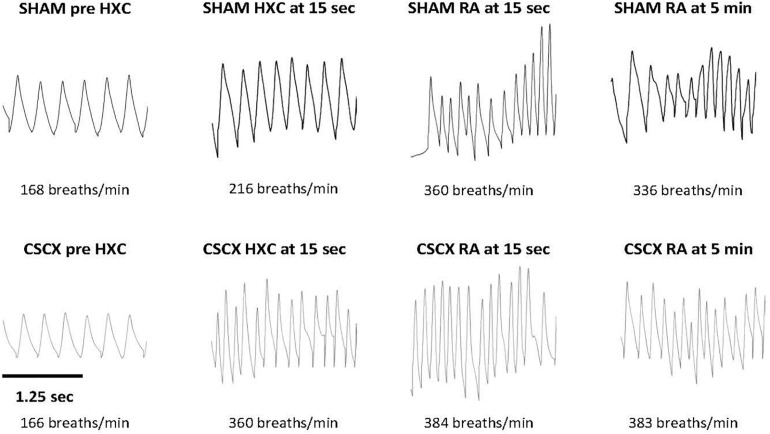
Example traces of respiratory waveforms during various stages of the experiment in a sham-operated (SHAM) mouse and in a mouse with bilateral cervical sympathetic chain transection (CSCX). A time bar (1.25 s) is shown in the bottom left of the figure. HXC, hypoxic gas challenge. RA, room-air.

Examples of respiratory waveforms during various stages of the experiment in a SHAM mouse and a CSCX mouse are shown in Figure 2. A time bar (1.25 s) is shown in the bottom left of the figure. Resting Freq (pre-HXC values) was similar in both groups of mice. The initial HXC response (HXC at 15 s) was far greater in the CSCX mouse (166–360 breaths/min = 194 breaths/min, +119%) than the SHAM mouse (168–216 breaths/min = 48 breaths/min, +29%). The roll-off values at the end of the 5 min HXC were similar between the two groups (data not shown) as were the increases in Freq immediately upon return to room-air (RA at 15 s, +131 and +114% for the SHAM and CSCX mouse, respectively) and at 5 min (RA at 5 min, +131 and +100% for the SHAM and CSCX mouse, respectively).

**FIGURE 3:**
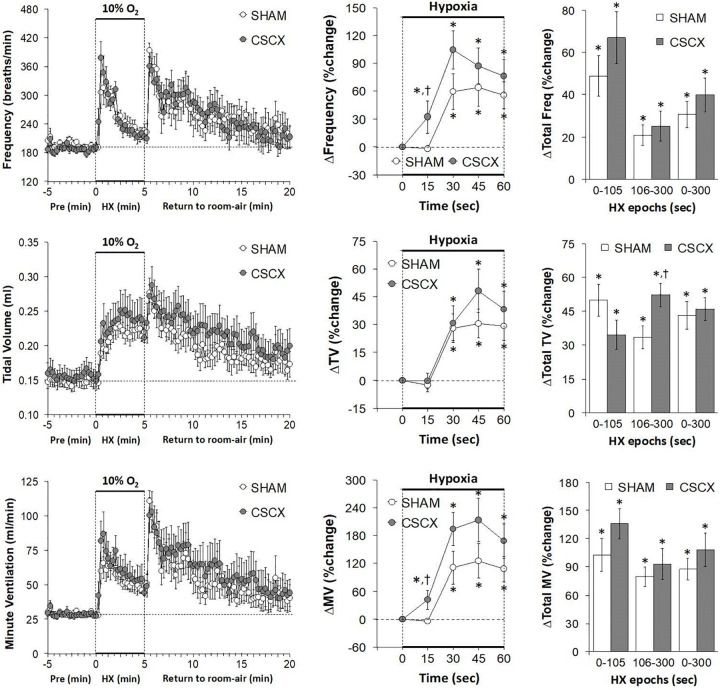
**(Left)** Frequency of breathing, tidal volume and minute ventilation values before, during and after a 5 min hypoxic (HX, 10% O_2_, 90% N_2_) gas challenge in sham-operated (SHAM) mice and in mice with bilateral transection of the cervical sympathetic chain (CSCX). **(Middle)** Responses (expressed as % of pre-values) during the first minute of HX challenge in SHAM and CSCX mice. **(Right)** Total responses (sum of all % changes from pre) during the first 105 s, between 106–300 s and 0–300 s. Data are expressed as mean ± SEM. *P < 0.05, significant response. ^†^*P* < 0.05, CSCX versus SHAM. There were 11 mice in the SHAM group and 12 mice in the CSCX group.

The changes in Freq, TV, and MV in SHAM and CSCX mice in response to the 5 min HXC and upon return to room-air are summarized in Figure 3. As seen in the top row of panels, exposure to HXC in SHAM mice elicited a typical initial increase in Freq that was subject to pronounced roll-off (left panel). The responses in CSCX mice were essentially similar compared to SHAM except that the rise in Freq was significantly higher at the 15 s time-point (middle panel). The total increases in Freq in the three designated epochs (0–105, 106–300, and 0–300 s) were similar in the SHAM and CSCX mice (right panel). The return to room-air elicited the expected dramatic increase in Freq in SHAM mice and CSCX mice (left panel), and the total room-air responses were similar in both groups (Table 2). As seen in the middle row of panels, exposure to the HXC elicited immediate and sustained increases in TV that were similar in most aspects in the SHAM and CSCX mice except that the total increase recorded during the 106–300 s epoch were higher in CSCX than SHAM mice (right panel). The return to room-air elicited the expected small initial increase in TV followed by gradual decline toward baseline in SHAM and CSCX mice (left panel). The total room-air responses were similar in both groups (Table 2). As seen in the bottom row of panels, exposure to HXC in the SHAM mice elicited a typical initial increase in MV that was subject to a pronounced roll-off (left panel). The MV responses in CSCX mice were similar except that the rise in MV was significantly higher at the 15 s time-point (middle panel). The total increases in MV in the three designated epochs were similar in the SHAM and CSCX mice. The return to room-air elicited the expected dramatic increase in MV in SHAM and CSCX mice (left panel). The total room-air responses were similar in both groups (Table 2).

**TABLE 2:** Total changes that occurred during the 15 min return to room-air.

**Parameter**	**SHAM**	**CSCX**
Number of mice	11	12
Frequency (%)	+33.0 ± 11.2	+39.4 ± 13.2
Tidal volume (TV, %)	+33.3 ± 7.1	+39.8 ± 7.4
Minute ventilation (%)	+87.1 ± 22.1	+105.3 ± 25.8
Inspiratory time (Ti, %)	−26.5 ± 5.5	−28.3 ± 5.0
Expiratory tme (Te, %)	+3.2 ± 7.9	+1.2 ± 8.2
+Te,%	+17.2 ± 3.4 (6)	+18.4 ± 3.1 (8)
−Te, %	−26.6 ± 4.0 (5)	−33.3 ± 5.1 (4)
Ti/Te, %	−25.3 ± 2.3	−25.2 ± 3.5
Ti/(Ti + Te), %	−18.9 ± 1.6	−19.0 ± 2.7
End inspiratory pause (EIP, %)	−12.9 ± 2.0	−10.7 ± 2.0
End expiratory pause (EEP, %)	+181 ± 29	+298 ± 61
Peak inspiratory flow (PIF, %)	+106 ± 19	+122 ± 21
Peak expiratory flow (PEF, %)	+92 ± 20	+128 ± 28
PIF/PEF, %	+13.5 ± 3.4	+6.9 ± 4.8
Expiratory flow at 50% exhaled TV (EF_50,_ %)	+89 ± 22	+114 ± 30
Relaxation time (RT, %)	−0.3 ± 6.4	−4.1 ± 7.3
+RT,%	+16.6 ± 3.0 (5)	+24.6 ± 7.9 (7)
−RT, %	−14.3 ± 3.7 (6)	−16.6 ± 3.0 (5)
Rate of achieving PEF (Rpef, %)	+27.1 ± 13.1	+21.9 ± 3.6
+Rpef,%	+61 ± 7 (6)	+21.9 ± 3.6* (5)
−Rpef, %	−14 ± 5 (5)	−23.6 ± 2.5 (7)
Inspiratory drive (TV/Ti, %)	+114 ± 21	+133 ± 24
Expiratory drive (TV/Te, %)	+59 ± 19	+78 ± 25
Rejection index (RI, %)	+227 ± 50	+200 ± 33
(Rejection index/frequency) × 100 (%)	+123 ± 27	+132 ± 28
Apneic pauses (Te/RT)-1 (%)	+8.2 ± 7.0	+13.8 ± 5.4
+(Te/RT)-1,%	+26.7 ± 6.5 (5)	+19.8 ± 3.3 (10)
−(Te/RT)-1, %	−7.2 ± 2.7 (6)	−16.5 ± 5.8 (2)

The data are presented as mean ± SEM. *P < 0.05, CSCX versus SHAM. The values in parentheses equal the number of mice in the sub-groups for Te, RT, Rpef and (Te/RT)-1.

### Hypoxic Challenges – Inspiratory Time and Expiratory Time

**FIGURE 4:**
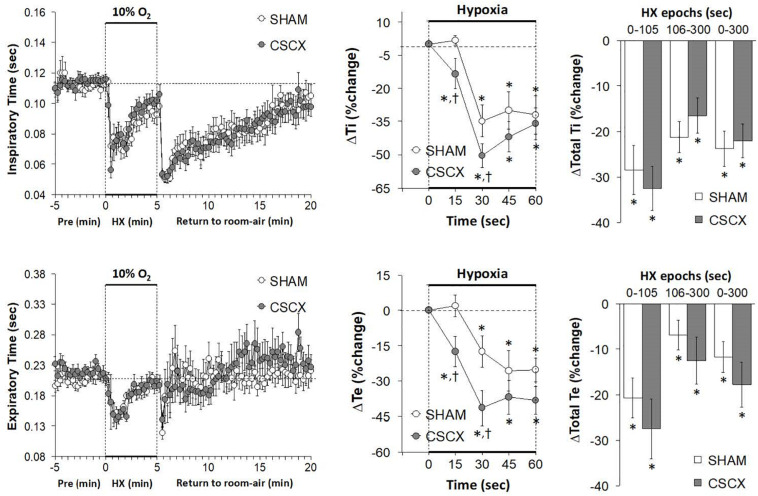
**(Left)** Inspiratory time (Ti) and expiratory time (Te) values before, during and after a 5 min hypoxic (HX, 10% O_2_, 90% N_2_) gas challenge in sham-operated (SHAM) mice and in mice with bilateral transection of the cervical sympathetic chain (CSCX). **(Middle)** Responses (expressed as % of pre-values) during the first minute of HX challenge in SHAM and CSCX mice. **(Right)** Total responses (sum of all % changes from pre) during the first 105 s, between 106–300 s and 0–300 s. Data are expressed as mean ± SEM. *P < 0.05, significant response. ^†^*P* < 0.05, CSCX versus SHAM. There were 11 mice in the SHAM group and 12 mice in the CSCX group.

The changes in Ti and Te values in SHAM and CSCX mice in response to the 5 min HXC and upon return to room-air are summarized in Figure 4. Exposure to HXC in SHAM mice elicited initial decreases in Ti and Te that were subject to roll-off (left panels). The decreases in Ti and Te occurred more rapidly in CSCX mice (middle panels), but the overall (total) responses for Ti and Te were similar in both groups (right panels). Return to room-air elicited a rapid transient decrease in Te, but a rapid and sustained decrease in Ti (left panels). The actual (left panels) and total room-air responses (Table 2) were similar in SHAM and CSCX mice. Additionally, the total Te responses upon return to room-air fell into two groups, those in which total Te fell and those in which Te rose (Table 2).

### Hypoxic Challenges – Inspiratory Time/Expiratory Time and Inspiratory Quotient

**FIGURE 5:**
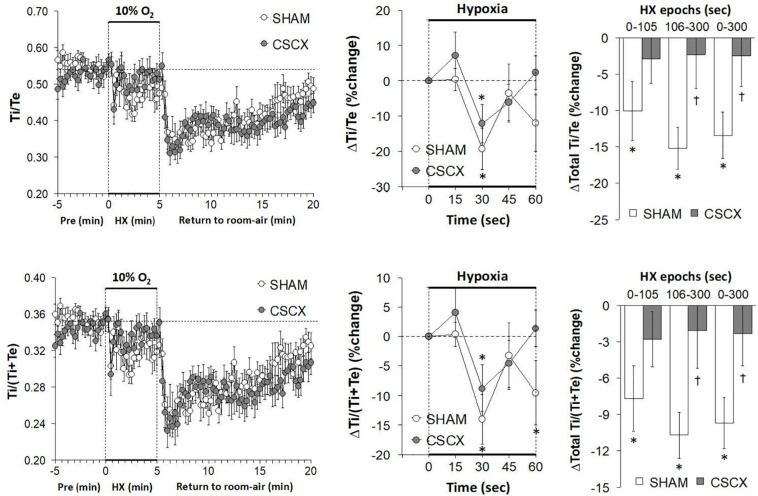
**(Left)** Inspiratory time/expiratory time (Ti/Te) and inspiratory time/(inspiratory time + expiratory time) ratios [Ti/(Ti + Te)] before, during and after a 5 min hypoxic (HX, 10% O_2_ and 90 %N_2_) gas challenge in sham-operated (SHAM) mice and in mice with bilateral transection of the cervical sympathetic chain (CSCX). **(Middle)** Responses (expressed as % of pre-values) during the first minute of HX challenge in SHAM and CSCX mice. **(Right)** Total responses (sum of all % changes from pre) during the first 105 s, between 106–300 s and 0–300 s. Data are expressed as mean ± SEM. *P < 0.05, significant response. ^†^*P* < 0.05, CSCX versus SHAM. There were 11 mice in the SHAM group and 12 mice in the CSCX group.

The changes in Ti/Te values and inspiratory quotients [Ti/(Ti + Te)] in SHAM and CSCX mice in response to the 5 min HXC and upon return to room-air are summarized in Figure 5. The resulting changes in Ti and Te during HXC (Figure 4) translated into minor changes in Ti/Te values and inspiratory quotients in SHAM mice (left and middle panels) that were nevertheless significantly smaller in the CSCX mice (right panels). As seen in the left panels, the return to room-air resulted in substantial decreases in Ti/Te values and inspiratory quotients that gradually returned toward baseline values in both groups. As seen in Table 2, the changes in total Ti/Te and Ti/(Ti + Te) values upon return to room-air were similar in the SHAM and CSCX mice.

### Hypoxic Challenges – End Inspiratory Pause, and End Expiratory Pause

**FIGURE 6:**
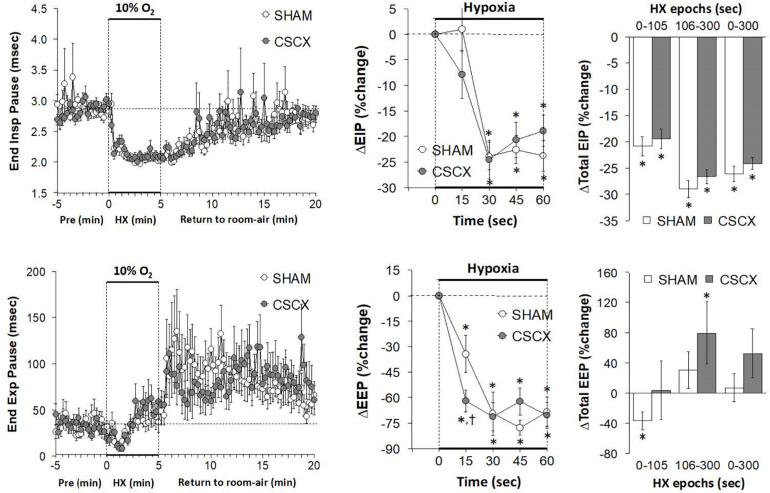
**(Left)** End inspiratory pause (EIP) and end expiratory pause (EEP) values before, during and after a 5 min hypoxic (HX, 10% O_2_, 90% N_2_) gas challenge in sham-operated (SHAM) mice and in mice with bilateral transection of the cervical sympathetic chain (CSCX). **(Middle)** Responses (expressed as % of pre-values) during the first minute of HX challenge in SHAM and CSCX mice. **(Right)** Total responses (sum of all % changes from pre) during the first 105 s, between 106–300 s and 0–300 s. Data are expressed as mean ± SEM. *P < 0.05, significant response. ^†^*P* < 0.05, CSCX versus SHAM. There were 11 mice in the SHAM group and 12 mice in the CSCX group.

The changes in EIP and EEP in SHAM and CSCX mice in response to the 5 min HXC and upon return to room-air are summarized in Figure 6. The hypoxic challenge elicited a prompt decrease in EIP in SHAM and CSCX mice (top left panel). The initial (top middle) and total responses (top right panel) were similar in SHAM and CSCX mice. The HXC elicited a fall in EEP of about 2 min in duration in SHAM and CSCX mice (bottom left panel). Then EEP quickly returned to baseline values in the SHAM mice during the remainder of the hypoxic challenge, but went above baseline in the CSCX mice. The initial decrease in EEP during the HXC occurred faster in the CSCX mice (bottom middle panel) and the total changes in EEP (bottom right panel) reflected the biphasic changes described above. Return to room-air resulted in a gradual recovery of EIP toward baseline values, but substantial and variable increases in EEP (left panels) for both groups. As seen in Table 2, the total EIP and EEP responses upon return to room-air were similar in the SHAM and CSCX mice.

### Hypoxic Challenges – Inspiratory Drive and Expiratory Drive

**FIGURE 7:**
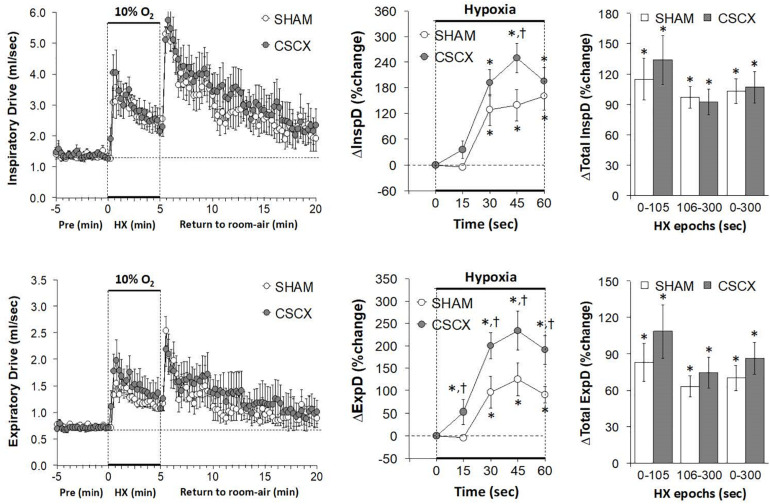
**(Left)** Inspiratory Drive (TV/Ti) and Expiratory Drive (TV/Te) before, during and after a 5 min hypoxic (HX, 10% O_2_, 90% N_2_) gas challenge in sham-operated (SHAM) mice and in mice with bilateral transection of the cervical sympathetic chain (CSCX). **(Middle)** Responses (expressed as % of pre-values) during the first minute of HX challenge in SHAM and CSCX mice. **(Right)** Total responses (sum of all % changes from pre) during the first 105 s, between 106–300 s and 0–300 s. Data are expressed as mean ± SEM. *P < 0.05, significant response. ^†^*P* < 0.05, CSCX versus SHAM. There were 11 mice in the SHAM group and 12 mice in the CSCX group.

The changes in inspiratory drive (TV/Ti) and expiratory drive (TV/Te) in the SHAM and CSCX mice in response to the 5 min HXC and upon return to room-air are summarized in Figure 7. The HXC elicited prompt and sustained increases in both inspiratory and expiratory drives (left panels) that occurred more rapidly in CSCX mice (middle panels) although the total responses were similar in both groups (right panels). Return to room-air elicited initial increase in both inspiratory and expiratory drives in SHAM and CSCX mice that gradually returned toward baseline (left panels). The total room-air changes were similar in both groups (Table 2).

### Hypoxic Challenges – Peak Inspiratory and Expiratory Flows

**FIGURE 8:**
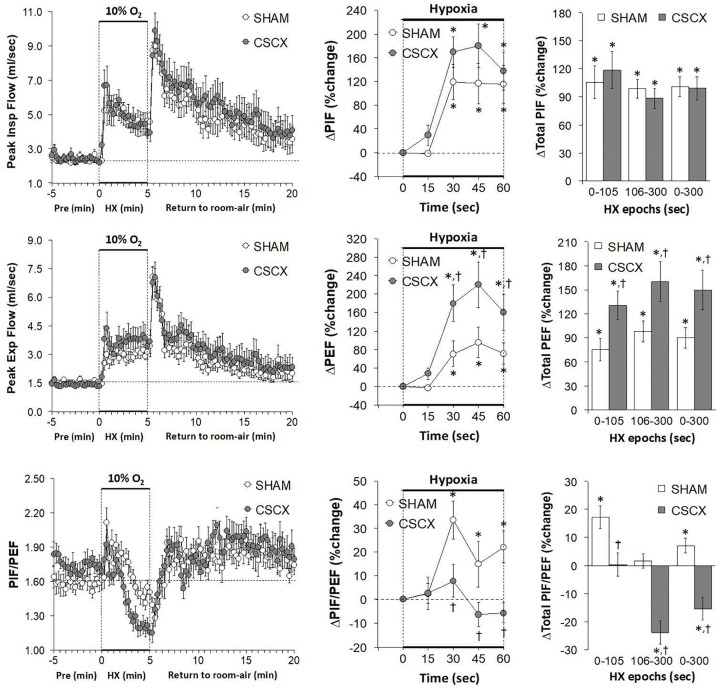
**(Left)** Peak Inspiratory flow (PIF) and Peak Expiratory Flow (PEF) values and PIF/PEF ratios before, during and after a 5 min hypoxic (HX, 10% O_2_, 90% N_2_) gas challenge in sham-operated (SHAM) mice and in mice with bilateral transection of the cervical sympathetic chain (CSCX). **(Middle)** Responses (expressed as % of pre-values) during the first minute of HX challenge in SHAM and CSCX mice. **(Right)** Total responses (sum of all % changes from pre) during the first 105 s, between 106–300 s and 0–300 s. Data are expressed as mean ± SEM. *P < 0.05, significant response. ^†^*P* < 0.05, CSCX versus SHAM. There were 11 mice in the SHAM group and 12 mice in the CSCX group.

The changes in PIF, PEF, and PIF/PEF ratios in the SHAM and CSCX mice in response to the 5 min HXC and upon return to room-air are shown in Figure 8. The HXC elicited prompt and sustained increases in PIF and PEF in SHAM and CSCX mice, with the PIF responses being of greater magnitude during the first half of the hypoxic challenge resulting in higher PIF/PEF ratios (left panels). The initial PIF responses during the HXC in the CSCX mice were similar to those in the SHAM mice, whereas the initial PEF responses were higher in CSCX mice, such that the expected increase in initial PIF/PEF ratios seen in SHAM mice did not occur in CSCX mice (middle panels). Total PIF responses to the HXC were similar in SHAM and CSCX mice, whereas the increases in PEF were higher in the CSCX mice compared to SHAM during each designated epoch (right panels). PIF/PEF ratios over the 106–300 and 0–300 s epochs were markedly lower in CSCX mice than in SHAM mice (bottom right panel). The return to room-air elicited initial increases in PIF and PEF in SHAM and CSCX mice, and the changes resulted in sustained increases in PIF/PEF ratios (left panels). Total PIF, PEF and PIF/PEF responses upon return to room-air were similar in both groups (Table 2).

### Hypoxic Challenges – EF_50_, Rpef, and Relaxation Time

**FIGURE 9:**
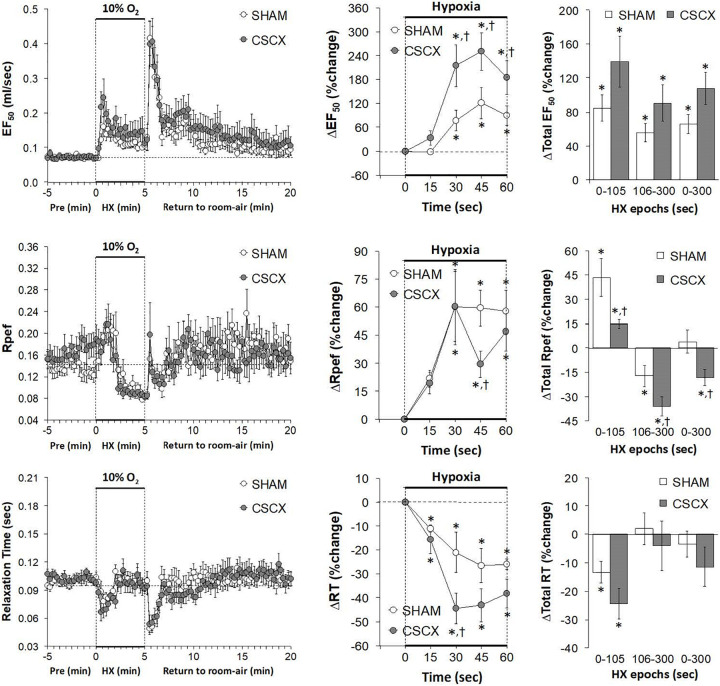
**(Left)** EF_50_, Rpef and Relaxation Time values before, during and after a 5 min hypoxic (HX, 10% O_2_, 90% N_2_) gas challenge in sham-operated (SHAM) mice and in mice with bilateral transection of the cervical sympathetic chain (CSCX). **(Middle)** Responses (expressed as % of pre-values) during the first minute of HX challenge in SHAM and CSCX mice. **(Right)** Total responses (sum of all % changes from pre) during the first 105 s, between 106–300 s and 0–300 s. Data are expressed as mean ± SEM. *P < 0.05, significant response. ^†^*P* < 0.05, CSCX versus SHAM. There were 11 mice in the SHAM group and 12 mice in the CSCX group.

The changes in EF_50_, Rpef, and RT values in the SHAM and CSCX mice in response to the 5 min HXC and upon return to room-air are summarized in Figure 9. HXC elicited a robust increase in EF_50_ that occurred more rapidly in the CSCX mice, although the total responses were similar in the SHAM and CSCX mice (top row: left, middle, and right panels). HXC-induced initial brief increases in Rpef in SHAM and CSCX mice that were followed by sustained decreases (middle row: left panel). These Rpef responses occurred more rapidly in the SHAM mice than CSCX mice at the 45 sec time-point (middle row: middle panel), and the responses during the 0–105 sec epoch were significantly smaller in the CSCX mice compared to SHAM, and significantly more reduced from baseline during the 106–300 sec epoch and overall 0–300 sec epoch (middle row: right panel). HXC elicited brief reductions in RT that occurred more rapidly in the CSCX mice than SHAM mice although the overall responses were similar in both groups (bottom row: left, middle, and right panels). The return to room-air elicited prompt increases in EF_50_ and Rpef that were associated with prompt decreases in RT (left panels). The overall room-air changes in EF_50_ were similar in both groups. The room-air changes in RT in the SHAM and CSCX mice fell into two categories of mice with roughly equal numbers, those in which RT was elevated and those in which RT was decreased. These two categories of changes were equivalent in the SHAM and CSCX mice. The changes in Rpef upon return to room-air also fell into two categories, roughly of equal numbers of mice, those in which Rpef was elevated and those in which Rpef was decreased. The values for those in which Rpef rose were significantly smaller in the CSCX mice than the SHAM mice (Table 2).

### Hypoxic Challenges – Rejection Index, Rejection Index/Frequency of Breathing, Apneic Pauses

**FIGURE 10:**
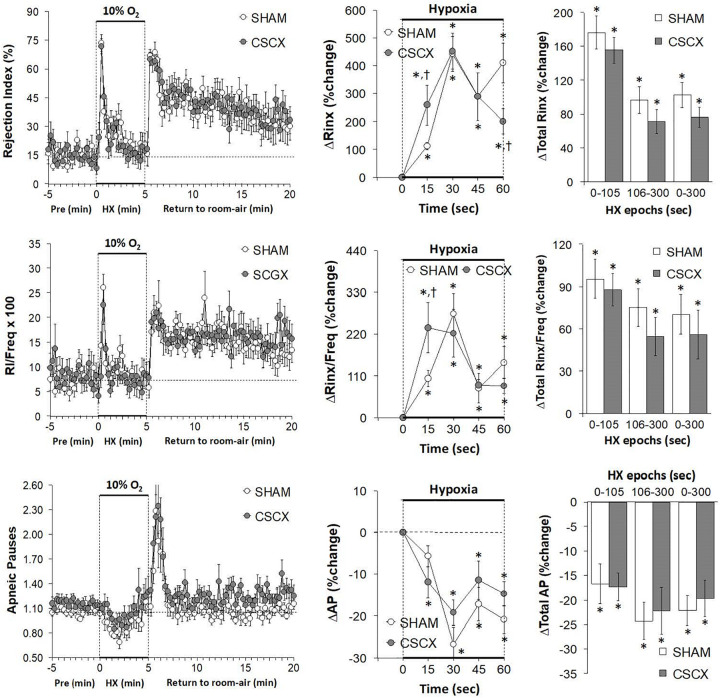
**(Left)** Rejection Index (RI, Rinx) values, RI/frequency ratios and Apneic Pauses (AP) before, during and after a 5 min hypoxic (HX, 10% O_2_, 90% N_2_) gas challenge in sham-operated (SHAM) mice and in mice with bilateral transection of the cervical sympathetic chain (CSCX). **(Middle)** Responses (expressed as % of pre-values) during the first minute of HX challenge in SHAM and CSCX mice. **(Right)** Total responses (sum of all % changes from pre) during the first 105 s, between 106–300 s and 0–300 s. Data are expressed as mean ± SEM. *P < 0.05, significant response. ^†^*P* < 0.05, CSCX versus SHAM. There were 11 mice in the SHAM group and 12 mice in the CSCX group.

The changes in RI, RI/Freq and Apneic Pause values in the SHAM and CSCX mice in response to the 5 min HXC and upon return to room-air are summarized in Figure 10. The HXC elicited immediate, but short-lived increases in RI and RI/Freq values that were not accompanied by increases in the numbers of apneic pauses, which on the contrary, showed relatively transient decreases in the earlier stage of the HXC (left panels). The increases in RI and RI/Freq occurred more rapidly in the CSCX mice, but the overall changes were similar in the SHAM and CSCX mice (top and middle rows: middle and right panels). The changes in apneic pauses during the HXC were similar in the SHAM and CSCX mice (bottom row). The return to room-air was associated with rapid and substantial increases in RI, RI/Freq and apneic pauses (left panel) that were similar in magnitude in the SHAM and CSCX mice (Table 2).

## Discussion

Due to the multiplicity and often opposing effects of sympathetic nerves/catecholamines in the carotid bodies (see Introduction), the question as to how the absence of functional sympathetic input to the carotid bodies and other structures controlling ventilatory processes would affect baseline parameters and the responses to HXC was not easy to predict. The major finding that many of the ventilatory responses (e.g., increases Freq, PEF and expiratory drive) during HXC occurred faster in CSCX mice suggests that activation of SCG sympathetic input to the carotid bodies of these mice normally blunts the initiation of these responses. Accordingly, it appears that loss of sympathetic input/catecholamine-induced suppression of carotid body activity (Kou et al., 1991; Pizarro et al., 1992; Prabhakar et al., 1993; Almaraz et al., 1997; Overholt and Prabhakar, 1999) out ways the loss of mechanisms which promote hypoxic responses including vasoconstriction in arterioles associated with glomus cells (Llados and Zapata, 1978; Eldridge and Gill-Kumar, 1980; Majcherczyk et al., 1980; Lahiri et al., 1981; Matsumoto et al., 1981; Gonsalves et al., 1984; Potter and McCloskey, 1987; Yokoyama et al., 2015). Deeper mechanistic insights would certainly come from studies designed to evaluate blood flow responses in the mouse carotid body and cerebral circulation in SHAM and CSCX mice during HXC and how systemic hemodynamic responses in these mice may influence the expression of the ventilatory responses.

### Ventilatory Responses During Hypoxic Gas Challenge

This study demonstrates that HXC in adult male C57BL6 mice elicits a complex array of ventilatory responses over and above what have been reported previously. In response to the HXC, the SHAM C57BL6 mice displayed an increase in Freq that was subject to roll-off and which was accompanied with decreases in Ti, Te, EEP, Rpef and RT that were also subject to roll-off. In contrast, the decrease in EIP was sustained throughout HXC. HXC was also associated with robust and sustained increases in TV, MV, PIF, PEF, EF_50_, and inspiratory and expiratory drives. The question arises as to why some ventilatory parameters in C57BL6 mice are subject to roll-off whereas others are not. First, it should be noted that the C57BL6 mouse is widely considered to be a “normal” healthy model to study the physiology of cardiorespiratory systems, and is used to generate genetically-engineered mice to study the mechanisms involved in cardiorespiratory-thermoregulatory control processes, including responses to HXCs (Tankersley et al., 2002; Campen et al., 2004, 2005; Palmer et al., 2013a,b; Tewari et al., 2013; Gaston et al., 2014). Despite considerable normal physiology, the C57BL6 mouse is of major interest to sleep-apnea researchers because it displays disordered breathing (irregular breathing patterns, including apneas and sighs) and cardiovascular disturbances during sleep and wakefulness, and shows disordered breathing upon return to room-air after exposure to HXC (Han and Strohl, 2000; Han et al., 2001, 2002; Tagaito et al., 2001; Yamauchi et al., 2008a,b,c, 2010; Getsy et al., 2014). The genetic (Tankersley et al., 1994, 2000, 2002; Tankersley, 2001, 2003; Han et al., 2001, 2002; Tagaito et al., 2001; Yamauchi et al., 2008b) and neurochemical processes (Tankersley et al., 2002; Price et al., 2003; Groeben et al., 2005; Yamauchi et al., 2008a,c, 2010; Moore et al., 2012, 2014), underlying the breathing patterns of C57BL6 mice, and the responses to HXC are well studied. The potential role of structural differences in the carotid bodies has also been studied (Yamaguchi et al., 2003, 2006; Chai et al., 2011). Nonetheless, evidence that the breathing patterns of C57BL6 mice and their responses to HXC have a strong genetic component has not posed an explanation as to why some ventilatory components, such as ventilatory timing (e.g., Freq and EEP) and mechanics (e.g., Rpef and RT) are subject to roll-off whereas other timing (e.g., EIP) and mechanics (e.g., PIF, PEF, and EF_50_) are not. Regardless of the explanation, understanding the importance of each of these ventilatory responses to HXC will help us better understand the processes by which breathing disorders occur in disease states and point to therapeutic strategies.

Resting ventilatory parameters (19 directly recorded or calculated variables) were similar in the SHAM and CSCX mice. This would suggest that (presumed) loss of input from the SCG to structures controlling breathing, such as the carotid bodies, upper airway and brainstem structures (see Introduction), do not obviously effect ventilatory timing or mechanics or the quality of breathing (e.g., occurrence of non-eupneic breathing events, such as apneic pauses) in C57BL6 mice. Again, the caveat is that these studies were performed only 4 days after CSCX, and it would seem possible that changes in baseline ventilatory performance would occur at longer post-CSCX time-points. Nevertheless, there were numerous important differences in the responses of CSCX and SHAM mice to the HXC. For example, the increases in Freq (and associated decreases in Ti, Te, EEP, and RT) and the increases in MV, inspiratory and expiratory drives, PEF and EF_50_ occurred more quickly in CSCX mice than in SHAM mice. Although both PIF and PEF increased during HXC in the CSCX mice, the PIF/PEF ratio fell because the rise in PEF exceeded that of the rise in PIF, and the PIF/PEF ratio fell more dramatically, and to a greater extent, in the CSCX mice because of the exaggerated rise in PEF in the CSCX mice. The overall (total) responses in the SHAM and CSCX mice to the HXC were similar to one another with some important exceptions. Specifically, the overall increases in TV during the latter half of the hypoxic challenge appeared greater in the CSCX mice, and as mentioned above, the PIF/PEF ratio fell and to a greater extent in the CSCX mice. Moreover, the total decreases in Ti/Te and respiratory quotient [Ti/(Ti + Te)] observed in the SHAM mice were absent in CSCX mice. These findings clearly demonstrate that CSC-SCG input to respiratory control structures influence the ventilatory responses to HXC in C57BL6 mice.

### Ventilatory Responses Upon Return to Room-Air

Following exposure to HXC, the return to room-air resulted in respiratory patterns that can be classified as short-term potentiation, in which ventilation remains elevated (Powell et al., 1998; Getsy et al., 2014) or post-hypoxic frequency decline, in which breathing frequency falls below baseline (Dick and Coles, 2000). Our C57BL6 mice displayed robust short-term potentiation upon return to room-air, accompanied by a substantial prolonged phase of disordered breathing (e.g., elevated rejection index). The mechanisms responsible for post-HXC disordered breathing have received considerable investigation, and at present, evidence is in favor of disturbances in central signaling (Wilkinson et al., 1997; Strohl, 2003) including, the pons area of the brainstem (Coles and Dick, 1996; Dick and Coles, 2000) rather than processes within the carotid bodies (Vizek et al., 1987; Brown et al., 1993), even though it is evident that carotid body chemoafferents play a vital role in the expression of disordered breathing such as, sleep apnea (Smith et al., 2003). The post-HXC responses were similar in our SHAM and CSCX mice, suggesting that diminished input to the SCG and (presumably decreased activity of SCG cells) do not have an obvious impact on the post-HXC responses, including the disordered breathing. The one exception was that total Rpef responses (positive rather than negative responders) (Table 2) after return to room-air were smaller in the CSCX mice than in the SHAM mice. This suggests that CSC-SCG activity is normally a positive factor in achieving maximal Rpef upon recovery from a HXC challenge in C57BL6 mice. The major differences with respect to the initial (i.e., first 60 sec of the HXC) changes in Freq, MV, EEP, rejection index (Rinx), and Rinx/Freq did occur during the first 15 s although other differences between the SHAM and CSCX groups occurred at 30 s for relaxation time and inspiratory drive; 45 s for Rpef; 15 and 30 s for Ti and Te; 30, 45, and 60 s for PEF, PEF/PIF and EF_50_; and 15, 30, 45, and 60 s for expiratory drive.

### Study Limitations

A limitation of this study is that it only provides a snap-shot of the temporal changes in ventilatory responses to HXC after bilateral CSCX. Therefore, it is imperative to study what the patterns of ventilatory responses to HXC would be at longer time-points post-CSCX. We chose to test the mice 4 days after CSCX surgery in order to compare with our findings related to the effects of bilateral removal of the SCG on HXC (see Conclusion). The 4-day recovery period was chosen for our other study as it would be the earliest time in which SCGX would result in virtually complete loss of sympathetic terminals within the carotid bodies (Mir et al., 1982; González-Guerrero et al., 1993; Ichikawa and Helke, 1993; Ichikawa, 2002). We have yet to establish whether this loss is true in mice, and also whether there is significant loss of sympathetic terminals in other structures, such as the brainstem. This presence/absence of intact terminals is vital since it is established that hypoxia elicits action potential/extracellular Ca^2+^-in*dependent* release of catecholamines from sympathetic nerve terminals (Schömig et al., 1987; Chahine et al., 1994; Kurz et al., 1995, 1996; Du et al., 1997). This evidence is especially relevant to our study since CSCX would presumably diminish activity of post-ganglionic neurons within the SCG without causing the degeneration of these post-ganglionic nerve terminals. Moreover, substantial data has shown that spinal cord damage, which markedly reduces pre-ganglionic outflow, does not necessarily eliminate all post-ganglionic sympathetic nerve activity (Meckler and Weaver, 1985; Qu et al., 1988; Stein and Weaver, 1988; McLachlan, 2007). Nonetheless, to our knowledge this has not been studied for T1–T4 spinal cord-CSC-SCG pathways. It would seem reasonable to suggest that since we transected the left and right CSC and therefore the pre-ganglionic fibers in these nerves, we could expect that (1) the activity of a post-ganglionic fibers emanating from the SCG including those to the carotid bodies would be markedly if not totally diminished, and (2) HXC would not be able to elicit centrally-mediated changes in SCG neuronal activity. However, it certainly remains possible that HXC directly activates post-ganglionic neurons denervated of pre-ganglionic input and/or more likely, direct release of neurotransmitters from sympathetic nerve terminals themselves as demonstrated in the heart and saphenous veins (Dart and Riemersma, 1991; Kamath et al., 1993; Chahine et al., 1994; Santos et al., 1996), if vesicular release mechanisms are intact.

## Conclusion

Our data show that under baseline (normoxic) environmental conditions, the potential loss of active sympathetic input to the carotid bodies (via CSCX-induced quiescence of post-ganglionic projections to the carotid body) does not have a noticeable effect on ventilatory parameters, which suggests that resting activity (e.g., neurotransmitter release) of carotid body glomus cells is not altered in a way that would lead to activation of carotid body chemoafferents and therefore enhancement of breathing. In contrast, our data shows that CSCX does alter the initial hypoxic ventilatory response and therefore suggests that diminished SCG input to the carotid bodies (or other targets, such as those in the brainstem) does influence the ability of glomus cells and/or other neuronal structures to respond to HXC. Planned studies involving bilateral transection of ganglioglomerular nerves (that project only to the carotid bodies and carotid sinus) will help to establish the relevant neuronal pathways/mechanisms. Overall, this novel data suggest that the CSC may normally provide inhibitory input to peripheral (e.g., carotid bodies) and central (e.g., brainstem) structures that are involved in the ventilatory responses to HXC in C57BL6 mice. Moreover, the results of our CSCX study lend support to the concept that a loss of CSC-SCG activity may be involved in the etiology of ventilatory disorders, such as sleep-disordered breathing. With respect to mechanistic insights provided by our data, it would be reasonable to assume that post-ganglionic sympathetic nerves innervating the ipsilateral carotid bodies (and other targets such as those within the brain) would be quiescent following CSCX as a result of the loss of pre-ganglionic input to the SCG. Whether the presumed diminution of sympathetic activity alters the expression of functional proteins (i.e., tyrosine hydroxylase, catecholamine-containing vesicles, and fusion proteins mediating vesicular exocytosis) within the sympathetic nerve terminals themselves and/or the target tissues, such as glomus cells in the carotid bodies needs to be addressed in future experiments. This is especially important since these post-transection adaptations in protein expression, while not being noticeable at rest (i.e., under normoxia) may have a direct effect on the ability of glomus cells (for example) to respond to the hypoxic challenge and/or secrete neurotransmitters. We have data that demonstrates that removal of the SCG has dramatically augmented effects on HXC compared to CSCX (unpublished observations). This raises important questions as to whether HXC may directly alter the activity of SCG neurons independently of the CSC input. One key question pertains to which of the SCG projections normally driven by the CSC are responsible for mediating the neuromodulatory effects of the CSC on the processes that drive ventilatory responses to HXC. As detailed in the Introduction section, the direct projections of the SCG to structures that control ventilation are extensive and include projections to the carotid bodies via the GGN. In order to better determine which of the post-ganglionic projections of the SCG regulate the ventilatory responses to HXC, we are currently planning to perform studies in mice in which the major post-ganglionic branches of the SCG, namely the internal carotid nerves, external carotid nerves and GGN are transected. We intend to perform these studies 4, 14, and 30 days post-transection in C57BL6 mice and in other strains, such as Swiss-Webster and A/J mice (Getsy et al., 2014) to determine temporal and genetic aspects of the role of the CSC-SCG complex in the control of ventilation and the responses to HXC.

The data in this study demonstrates that the primary effect of CSCX appears to be changes in the immediate responsiveness to the HXC. For example, the increases in Freq (and associated decreases in Ti, Te, and EEP), MV, expiratory drive, and rejection index occurred more rapidly in CSCX mice than SHAM mice (changes at 15 sec were significant in CSCX mice but not SHAM mice and between-group differences for expiratory drive were maintained at 15, 30, 45, and 60 s). In contrast, between-group differences in the responses of relaxation time, Rpef, PIF, PEF, PEF/PIF, EF_50_, and inspiratory drive, were evident at 30, 45, or 60 s with between-group differences for PEF, PEF/PIF, and EF_50_ being evident at 30, 45, and 60 s. Additionally, it is important to remember for the interpretation of the effects of CSCX that it was evident that the initial increases in TV and PIF during HXC were similar in SHAM and CSCX mice. Taken together, it is apparent that the loss of post-ganglionic SCG input to structures, such as the carotid body and brainstem, has a strong impact on ventilatory performance in C57BL6 mice. The findings that the decreases in Ti and Te were larger in the CSCX mice than the SHAM mice suggests that SCG input to neural structures regulating ventilatory timing events equally affect inspiratory and expiratory control processes. However, with respect to flow parameters it was evident that (a) the increases in TV were similar in SHAM and CSCX mice, (b) the responses of PIF and inspiratory drive in CSCX mice were minimally different from SHAM mice, whereas the changes in PEF, EF_50_, PEF/PIF and expiratory drive were substantially different between the groups. As such, CSCX appears to have much more of an influence on PEF than PIF. Whether this pattern of effects is due primarily to altered carotid body function must await more definitive studies in which, for example, the effects of bilateral GGN transection are investigated.

The data in the present manuscript and in that of our recent study which investigated the effects of bilateral SCGX (unpublished observations) provides the beginning of understanding how the loss of pre-ganglionic and/or post-ganglionic fibers in the CSC-SCG complex affect resting ventilatory parameters and the responses to HXC. On-going studies will extend our investigations by (1) determining how expression of proteins in sympathetic nerve terminals (e.g., tyrosine hydroxylase, fusion proteins, and vesicular stores of norepinephrine) and carotid body glomus cells (e.g., tyrosine hydroxylase, voltage-gated Na^+^, K^+^, and Ca^2+^-channels) change after CSCX, and (2) differentiating the effects of sympathetic input to various areas by performing ventilatory studies in mice with (a) transection of the left and right internal carotid nerves (a major post-ganglionic SCG trunk), (b) transection of the left and right external carotid nerves (the other major post-ganglionic SCG trunk), and (c) transection of the left and right ganglioglomerular nerves (a branch of the external carotid nerve), which projects only to the carotid bodies and carotid sinus.

## Data Availability Statement

The raw data supporting the conclusions of this article will be made available by the authors, without undue reservation.

## Ethics Statement

The animal study was reviewed and approved by Case Western Reserve University Institution’s Animal Care and Use Committee.

## Author Contributions

PG, GC, Y-HH, and SL conceived and designed the study. PG performed the mouse surgeries. PG and GC performed the plethysmography studies. PG and SL analyzed the data and prepared the figures. All authors contributed to writing the manuscript, and revised, read, and approved the final version of the manuscript.

## Conflict of Interest

The authors declare that the research was conducted in the absence of any commercial or financial relationships that could be construed as a potential conflict of interest.

## Footnotes

1 https://www.nap.edu/catalog/5140/guide-for-the-care-and-use-of-laboratory-animals

2 https://www.datasci.com/products/buxco-respiratory-products/finepointe-whole-body-plethysmography

## Abbreviations

CSC: cervical sympathetic chain
CSCX: cervical sympathetic chain transection
SCG: superior cervical ganglion
SCGX: superior cervical ganglionectomy
HXC: hypoxic gas challenge
Freq: frequency of breathing
TV: tidal volume
MV: minute ventilation
Ti: inspiratory time
Te: expiratory time
EIP: end inspiratory pause
EEP: end expiratory pause
PIF: peak inspiratory flow
PEF: peak expiratory flow
RT: relaxation time
EF_50_: expiratory flow at 50% expired total volume
Rpef: rate of achieving peak expiratory flow.
